# Three Major Deficiency Diseases Harming Mankind (Protein, Retinoid, Iron) Operate Under Tryptophan Dependency

**DOI:** 10.3390/nu17152505

**Published:** 2025-07-30

**Authors:** Yves Ingenbleek

**Affiliations:** Laboratory of Nutrition, Faculty of Pharmacy, University of Strasbourg, 67401 Strasbourg, France; nutri.yvingenbleek@protonmail.com; Tel.: +33-04-67-74-87-17

**Keywords:** lean body mass, transthyretin, retinol-binding protein, iron deficiency, tryptophan deficiency, protein malnutrition, inflammatory disorders, immune reactions

## Abstract

This story began half a century ago with the discovery of an unusually high presence of tryptophan (Trp, W) in transthyretin (TTR), one of the three carrier proteins of thyroid hormones. With the Trp-rich retinol-binding protein (RBP), TTR forms a plasma complex implicated in the delivery of retinoid compounds to body tissues. W has the lowest concentration among all AAs involved in the sequencing of human body proteins. The present review proposes molecular maps focusing on the ratio of W/AA residues found in the sequence of proteins involved in immune events, allowing us to ascribe the guidance of inflammatory processes as fully under the influence of W. Under the control of cytokine stimulation, plasma biomarkers of protein nutritional status work in concert with major acute-phase reactants (APRs) and with carrier proteins to release, in a free and active form, their W and hormonal ligands, interacting to generate hot spots affecting the course of *acute* stress disorders. The *prognostic inflammatory and nutritional index* (PINI) scoring formula contributes to identifying the respective roles played by each of the components prevailing during the progression of the disease. Glucagon demonstrates ambivalent properties, remaining passive under steady-state conditions while displaying stronger effects after cytokine activation. In developing countries, inappropriate weaning periods lead to toddlers eating W-deficient cereals as a staple, causing a dramatic reduction in the levels of W-rich biomarkers in plasma, constituting a novel nutritional deficiency at the global scale. Appropriate counseling should be set up using W implementations to cover the weaning period and extended until school age. In adult and elderly subjects, the helpful immune protections provided by W may be hindered by the surge in harmful catabolites with the occurrence of *chronic* complications, which can have a significant public health impact but lack the uncontrolled surges in PINI observed in young infants and teenagers. Biomarkers of neurodegenerative and neoplastic disorders measured in elderly patients indicate the slow-moving elevation of APRs due to rampant degradation processes.

## 1. Introductory Steps

**Figure 1:**
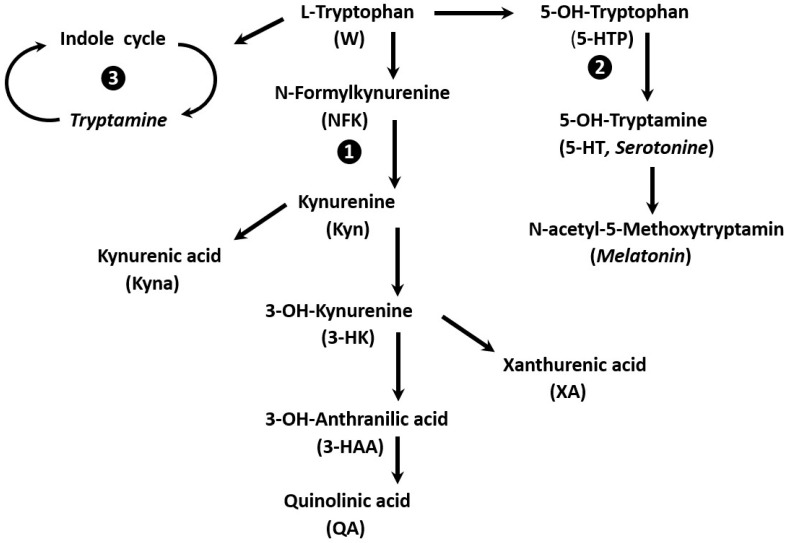
Schematic representation of the main metabolic pathways of W. L-W is a natural EAA found in human and animal tissues. Dietary L-W is absorbed by the intestinal mucosa and circulates in the bloodstream mainly bound and transported by ALB with minor values remaining in free form. Three main W pathways are identified: ❶ Quantitatively, the Kyn pathway is endowed with the largest representation under the control of several rate-limiting enzymes (indoleamine-2,3-dioxygenase, W-2,3-dioxygenase, kyn-3-monooxygenase and W-hydroxylase). Most Kyn derivatives may cross the BBB and control brain function, degeneration and apoptosis. The conversion of 3-HAA to the neurotoxic QA is implicated in deterioration of cognitive functioning and Alzheimer’s disease. ❷ W may be converted into 5-HTP and further into 5-OH-tryptamine (serotonine) that take place in both the gut and the brain and is stored in the platelets, being involved in vasoconstrictive processes and stimulation of smooth muscular cells. Functioning of serotonine is depending on a large set of cellular receptors operating in many organs, including in cerebral tissues where it may be converted to melatonin in the pineal gland, assuming the regulation of circadian rhythms (sleep-wakefulness and light-dark cycles). ❸ The intestinal flora metabolizes W to indole and derivatives, including skatole and tryptamine. The roles played by these derivatives is the maintenance of intestinal immune homeostasis and preservation of microbiota.

Neuropathologists have recently published authoritative reviews [1,2] providing detailed information on most metabolic disorders of W recorded throughout recent decades. Continuing gains in knowledge have been registered, and three main pathways are now clearly delineated, as summarized in Figure 1. The *first* converts the bulk of dietary W (95%) into kynurenine (Kyn), which may be transformed into bioactive compounds using crucial enzymes (indoleamine-2,3-dioxygenase IDO1 and IDO2) in the regulation of immune responses [3]. The *second* pathway converts W into the vasoconstrictive serotonin molecule (5-hydroxytryptamine, 5-HT), which may undergo elongation to melatonin (*N-*acetyl-5-methoxytryptamine) located in the pineal gland and involved in controlling sleep–wake cycle activity [4]. The *third* pathway attracts attention to the gut microbiota, converting W into indole, tryptamine, skatol, and derivatives to maintain the intestinal homeostasis and activity of the gut–brain axis [5]. In neurodegenerative disorders, the accumulation of neurotoxic molecules may occur in brain tissues, as shown for quinolinic acid (QA) overexpressed in the presence of Alzheimer’s amyloid plaques [6]. Several neurological or even neuropsychiatric disorders may develop, such as postpartum depression, with its symptoms of insomnia, anxiety, confusion, and even suicidal tendencies [7] sometimes being associated with glioblastoma [8]. These latter cerebral complications are currently attributed to increased values of Kyn pathway products with the downregulation of serotonin and melatonin components. Specific W metabolites contribute to the occurrence of neurodegenerative disorders such as Alzheimer’s, Parkinson’s, and Huntington‘s diseases [9]. These patients may also demonstrate increased vulnerability to long COVID [10].

Taken together, these investigations document the view that abnormalities of W metabolism may deteriorate the clinical conditions of middle-aged men and women and of elderly subjects living primarily in developed countries. Considerable research efforts are underway to establish pharmacological drugs capable of inducing inhibitory effects on the key enzymes dysregulating W pathways. These approaches constitute promising therapeutic targets, using, in order of priority, immunology, enzymology, and neurobiology as the main research tools. These major advances nevertheless depart from the basic clinical and pathophysiological patterns encountered in developing countries where W deficiency appears to more specifically target pregnant women and preschool children. These research approaches engaged in disentangling the intricate W-dependent clinical deficiencies at the worldwide level preferentially refer to medical disciplines related to nutrition and endocrinology. The below review attempted to collect the available data from developing areas counterbalancing the prevailing roles played by W in westernized societies.

## 2. Historical Background

In previous centuries, kwashiorkor (KW) and marasmus (MR) were the two extreme poles of dietary deficiencies commonly harming infant population groups in most developing countries. C. Williams was the first to pinpoint the roles played by vegan diets during inappropriate weaning periods [11]. Her primary publication was supported by the World Health Organization (WHO) [12]. The clinical observations of malnourished children launched intense research efforts aiming to identify the severity of their nutritional disorders and to sustain relevant rehabilitation endeavors. The measurement of serum albumin (ALB), carried out at the University of Witwatersrand (South Africa), was rapidly recognized for its clinical usefulness [13]. During the two following decades, the ALB biomarker saw universal dissemination in developing areas, which also stimulated the detection of subclinical and unrecognized forms of protein malnutrition prevailing in Western medical [14] and surgical [15] wards.

In the early 1970s, a large nutritional and endocrine survey was undertaken at the University of Dakar (Senegal, West Africa), in close collaboration with the University of Louvain (Belgium), aiming at measuring most of the available functional thyroid parameters in KW and MR Senegalese children. At that time, the third and last carrier protein of thyroid hormones was discovered [16], identified as a small tetrameric peptide migrating just in front of the huge ALB peak in usual electrophoretic systems, hence its initial designation as thyroxine-binding prealbumin (TBPA). The main conclusions of this African survey were published in 1972 [17], showing that both TBPA and ALB indices demonstrated similar declining and/or rising trends in the course of malnutrition but with higher sensitivity and steeper slopes for the former over the latter owing to significant differences in biological half-lives. Some years earlier, German workers [18] described the unusually high TBPA richness in W, a chemical peculiarity suggesting that the carrier protein might play unforeseen roles in human biology. Their proposal escaped attention for decades and was even subjected to disapproval [19]. The complete amino acid (AA) sequence of TBPA was cleared up some years later [20], showing that the protein was also involved in the transport of its companion retinol-binding protein (RBP) carrying its retinol ligand [21]. As a result, the TBPA symbol was removed from the prevailing scientific literature and replaced with the new *transthyretin* (TTR) denomination [22]. The trimolecular TTR-RBP-retinol edifice is currently designated under the retinol circulating complex (RCC) acronym [23], recalling that it is in charge of delivering both thyroid and retinoid compounds to body cells.

## 3. The Discovery of LBM, TTR, and RBP in Healthy Subjects

Schematically, the human body may be divided into two major compartments, fat mass (FM) and lean body mass (LBM). The latter collects most of the total body nitrogen (N) comprising a composite agglomeration of all organs and tissues involved in structural and specific functional properties. Under healthy conditions, protein homeostasis is the net result of balance between protein synthesis and protein breakdown. Two main metabolic pathways contribute to this recycling process. The *first* concerns about 2/3 of proteins undergoing endogenous breakdown, yielding their 20 constitutive AAs that may be recovered as new protein syntheses. The remaining 1/3 of proteins are excreted in the urinary output in the form of lost catabolites (urea, ammonia, creatinine) with a minor N fraction released as free AA residues. The *second* recycling process is sustained by the regular dietary intake of animal and/or plant proteins ensuring the provision of nine essential AAs (EAAs) as demonstrated throughout the pioneer studies performed by Rose [24]. All EAAS are of vital importance, and the dietary deprivation of a single one of them may lead to the inhibition of mRNA levels in cultured hepatoma cells, resulting in the abrogation of both ALB and TTR synthesis [25].

In body composition approaches, two studies emerge as having provided major global and detailed information on LBM in health and disease. The earliest investigations were launched by Forbes at the University of Rochester (USA) using dual-energy X-ray absorptiometry for the detection of ^40^K [26]. This tiny ß-radioactive K isotope is characterized by close interchangeable turnover rates with the naturally occurring ^39^K, in turn narrowly correlated to N (nitrogen values) [27] in human tissues. Forbes’s approaches comprised seven clinical studies stratifying the whole human lifespan, allowing Forbes to collect LBM data from birth to a very old age [26]. The following studies were undertaken by Bienvenu et al., aiming to measure plasma TTR concentrations in a very large population group of 68,720 healthy North American citizens of all ages and both sexes [28]. The trajectories of LBM data and TTR concentrations were shown to be closely superimposable regardless of age and gender [29]. There exist high correlations (r = 0.64) between TTR plasma values and LBM, meaning that the nutritional biomarker works as a universal tool, allowing us to grade the residual LBM capacity to reflect disease burden [30]. The close LBM-TTR correlation is mainly explained by the fact that TTR remains confined within a stable intravascular space, subjected to neither extravasation nor intestinal [31] or renal losses [32]. The LBM-ALB correlation appears to be slightly weaker (r = 0.52) [30] owing to the fact that ALB has a long T ½ of 19 days, indicating that it is slowly responsive to LBM fluctuations [29]. RBP-retinol was once considered a (excellent) possible candidate to fulfill the scoring task [33] but it has been downgraded in recognition of the heterogeneity of all RBP molecular species in body fluids and tissues [34]. RBP is indeed regarded as the main carrier protein in charge of retinol transport from liver to peripheral tissues using a number of specific membrane, cytosolic, and nuclear receptors to fine-tune the delivery of a dozen distinct retinoid derivatives to cerebral, retinal, cutaneous, adipose, renal, muscular, and fetal tissues [34]. In addition, significantly increased RBP and retinol values may be leaked via urinary output during acute infections [35]. As a result, plasma ALB is now classified, just after TTR, as the second more pertinent biomarker to represent LBM fluctuations. This is supported by a recent comprehensive Danish survey closing an old debate confirming that plasma ALB values more faithfully reflect fluctuations in nutritional protein states over those produced by inflammatory disorders [36].

## 4. Energy and Protein Requirements in Healthy Subjects

In healthy human subjects, the total basal body heat production, usually defined as resting energy expenditure (REE) is shared among organs and tissues pertaining to LBM: 24.3% for brain tissues, 19.9% for liver and 19.4% for skeletal muscle [37], 10% for intestinal mucosa [38], and 7% for thymoleukocytic tissues [39]. In the case of chronic overfeeding, the carbohydrate compartment may be subjected to upgrowth sequestration of glycogen in liver and muscle mass, whereas the fat compartment may similarly undergo storage and lipid reserves in several body tissues. LBM escapes these uprising trends owing to the fact that any dietary surplus in *N-*compounds is subjected to instant catabolism, implying that plasma TTR works as a nutritional indicator faithfully reflecting LBM fluctuations. There exists in the literature a large body of illnesses initially harming only one single organ or function, as seen in pancreatitis [40] or solid tumors [41]. These clinical conditions downregulate the TTR value, which works as a punctuation marker for information on the stress–induced protein losses of the inflicted organ. Such morbid processes are usually associated with up and down TTR fluctuations, showing that LBM may undergo *N-*recovery or depletion processes influencing liver secretory rates of plasma TTR, which emerges as the ultimate biomarker of LBM and is regarded as a cornerstone of bodybuilding [29,30]. We postulate the existence of still unknown metabolic pathways and centrally mediated molecular mechanisms governing the balance between protein accretion and breakdown in the whole body. A good example of such likely connections is provided by coeliac patients submitted to gluten-free diets allowing the restoration of their duodenal villous thinness and generating concomitantly increasing TTR plasma values [42].

## 5. LBM and W in Inflammatory Disorders

This section attempts to collect recent data recorded on W metabolism and on the multiple and complex relationships linking TTR and RBP to other W-rich molecules. These approaches have narrow implications, with most mediators and reacting substrates taking part in the development of immune responses affecting healthy subjects and malnourished patients. Over the last two decades, considerable research efforts were undertaken to clarify the evolutionary protein engineering processes that produced the prebiotic environment required to reach the emergence of the 20 AAs that constitute modern nutrition. It has been suggested that millions of years were necessary to create a reduced primordial genetic code, leading to the surge of some primitive AAs [43]. Many other AAs, including the so-called branched-chain AAs (BCAAs, i.e., leucine, isoleucine, and valine), arose later in the primordial soup [44]. The more complex sulfur-methionine (S) and both aromatic EAAs, phenylalanine (F) and (Trp) W, were the last to emerge [45]. The number of codons alone supports that S, F, and W were introduced during the third and last evolutionary step of the genetic code [46].

### 5.1. The Primary LBM Domain

Table 1 lists organs and tissues comprising most AAs and proteins produced by the primary and secondary genetic codes, with the latter constituting the bulk of branched-chain AAs mainly confined to the muscle mass sequestering about 35% of all BCAAs from the body. Large publications have documented the complex roles played by the three constitutive BCAAs through the mediation of their glycogenic and ketogenic derivatives in the regulation of normal growth, energy expenditure, glycogenesis, and glycogenolysis via oxidization, transamination, or conversion processes in the liver, heart, kidneys, intestine, and adipocytes [47]. Table 1 documents some main structural characteristics of the four hormonal peptides regulating growth and energy expenses in normal subjects. The first three peptides, HGH [48], insulin [49], and IGF-1 [50], constitute a homogeneous group poorly responsive to alterations imposed by inflammatory disorders, whereas glucagon [51] must be treated separately for the reasons below exposed. The AA sequences of insulin and IGF-1 are totally deprived of W, while HGH comprises a single W molecule located within a 191 AA sequence. (Trp) W is the least abundant molecule measured in all proteins pertaining to the human body, with a median value usually turning around 1.1% in most AA protein sequences. We sustain hereunder the view that the potential capacity for W to participate in immune reactions depends on the ratio linking the number of W residues versus the total number of AA molecules defining the sequence of each harboring protein. This proposal of a molecular map shows that a W/AA ratio less than 1 implies a reduced capacity to exert W-induced effects on an inflammatory environment. Opposite results are recorded when the W/AA ratio is more than 1, indicating that W may benefit from increasing opportunities to influence the course of inflammation in proportion to its elevation in the core of injured tissues. The data in Table 1 show that, owing to the W/AA ratio equaling zero, insulin [49] and IGF-1 [50] do not directly take part in immune responses, whereas HGH [48], with a low 0.5 value, may minimally participate in stress reactions. Glucagon [51] is a small molecule comprising only 29 AAs with one single W molecule, hence yielding a W/AA ratio exceptionally elevated at 3.45 that should, once activated, trigger strong W stimulatory impulses. The AA sequence of a protein should be regarded as a valuable source of insight into its functionality. To the best of our knowledge, no proteins have been found in the human body that might overtake the W/AA ratio of 4% nearly reached by glucagon. We postulate that exceeding this top 4% level should not be compatible with adaptive resilience or molecular survival. An interesting study undertaken in Jamaican children suffering from KW but devoid of infectious complications provided useful clarifications concerning the behavior of the main hormonal parameters, notably for those of glucagon [52]. Malnourished infants admitted to the Caribbean pediatric ward demonstrated a loss of body weight (BW), reduced turnover rate of body proteins, and downregulated kidney output of urea and N-catabolites. Both insulin and glucagon plasma values are initially decreased but reveal progressive restoration during catch-up growth resulting from dietary rehabilitation, reflecting an increased metabolism leading to progressive tissue repair. The promotion of new protein syntheses is achieved through the anabolic activities of insulin prevailing over the catabolic properties of glucagon, allowing the maintenance of unaltered blood sugar levels throughout the investigation [52]. This survey shows that, in the absence of inflammation and cytokine induction, glucagon provides a passive contribution to the maintenance of a balanced anabolic/catabolic equilibrium and to the stabilization of the neuroendocrine HGH, somatomedins, insulin, and glucagon axis.

The W/AA ratio attributed to insulin and IGF-1 indicates that these mediators do not take part in immune responses and maintain neutral and steady-state positions. HGH has marginal immune potency, whereas glucagon reveals extremely high capacities expressed only after cytokine activation.

### 5.2. The Second LBM Domain

The influence of W on the regulation of inflammatory disorders (Table 2) mainly covers all hormonal mediators involved in pathophysiological aspects associated with prevention and defense against aggressive agents, together with the containment of injury and repair processes. This area is typically a reserved domain for W, as it is shown that insufficient dietary intake of this EAA and/or its defective conversion to L-kynurenine imposed by cytokine-induced inhibition of indoleamine 2,3-dioxygenases may dysregulate NK-cell function and activate T-cells to apoptosis [53,54]. Table 2 shows some characteristics of the main compounds participating in a direct manner in the W-mediated events inducing biological reactions in the course of inflammation. In all circumstances, activation of macrophages by several cytokines (interleukins-1 and -6, tumor necrosis factor-α, interferon-γ) triggers TTR and RBP breakdown, hence releasing substantial amounts of W molecules as well as their thyroxine and retinol ligands in physiologically active free forms [55]. The surge of such free endocrine pools is many times larger than those recorded in healthy people, and their peak levels are reached during the first days after initial impact [55]. Following the law of mass action [56], the amplitude of this transient hyperthyroxinemia and hyperretinolemia is proportionate to the decrement between pre- and post-stress TTR and RBP levels. This implies that in the case of pre-existing malnutrition marked by low RCC values, this first line of defense may be blunted. During sepsis and fever, accelerated turnover of freed thyroid hormones works as a modulator of immune activities [57], notably bolstering the multiple roles played by AGP [58]. In the same inflammatory context, free retinol may antagonize a number of tissue inadequacies and promote organ damage repair [59]. Ongoing studies show that the released ligands and their synthesis products may develop interconnected cross-relationships likely assigned to provide better adaptive immune reactions [60]. ALB, secreted by the liver, consists of a nonglycosylated polypeptide comprising 585 AAs with one single W^214^ residue and 34 Cys AAs forming 17 disulfide S-S bonds, whereas only one single Cys^34^ remains in free and redox-active form [61]. ALB is therefore endowed with a very low W/AA score (0.17), but this apparent handicap is largely overcompensated by the fact that, in healthy subjects, the huge ALB molecular (body) mass is distributed in an extracellular space of about 5 L. The normal ALB plasma concentration is approximately 45 g/L, mainly sequestered in intravascular and intraventricular cerebrospinal fluid (CSF) spaces (including those of cerebrospinal fluid (CSF)). Critically ill patients incur survival risk when ALB values drop to 24.5 g/L [62], meaning that in the most severe cases of malnutrition/inflammation, ALB may still safeguard nearly half its W^214^ reservoir to face further immune challenges. ALB is also vitally important as an anti-oxidative agent [63] representing about 70% of the plasma free radical trapping capacity, working mainly through its multiple binding sites and transport properties. The largest proportion of W-bound ALB (up to 75%) may be released in free form to reach the blood–brain barrier (BBB) from where it may be taken up by the brain [64]. During the course of infection, AAs and inflamed tissues are decomposed as catabolites into cellular fragments and molecular debris circulating in body fluids. Cytokines and hyperglucagonemic states work in concert to set up febrile and violent outbursts clearly distinct from the passive pictures prevailing in the absence of cytokine and hormonal impulses. Charlton et al. were among the first researchers demonstrating convincingly that glucagon is the pivotal hormone dedicated to the removal of catabolic waste material [65]. Two major excretory pathways have been determined: kidney studies have displayed increased urinary output of urea and *N-*catabolites [66], and dog experiments have shown increases in hepatic AA uptake and extraction, resulting in a 17% decrease in total plasma AAs [67]. The roles played by glucagon appear to be complementary to the cleansing work completed by ALB as anti-oxidative leading molecule.

The numbers encircled in Table 2 indicate the W units inserted into the AA sequence of each corresponding molecular species. Both TTR and RBP biomarkers are high in W, enabling cytokine breakdown, triggering the release of their thyroxine and retinol ligands in physiologically active form, together with a freed W pool working in concert with W residues discharged from AGP and CRP decay. ALB does not directly participate in immune activities, but its large amount of MM circulating in body fluids serves as a dispenser of non-covalently bound W residues to face peripheral requirements. HGB and TF molecules handle all complex immune aspects dysregulating iron metabolism under the dependency of W availability.

### 5.3. Contributory Roles Played by Major APRs

Alpha-1-acid glycoprotein (AGP) is one of the two main APRs [68] secreted by the liver, displaying multiple modulatory activities implicated in immune responses to inflammatory disorders [72]. AGP is heavily glycosylated (42%), bearing different carbohydrate chains covalently bound to five asparagine residues. In the course of inflammation, the kinetics, uptake, half-life, and proper catabolism of each AGP moiety may be altered [68]. The plasma physiological level of AGP is usually situated below 0.7 g/L, which may be upgraded two or three times during the sepsis process. It is worth noting that, among the multiple ligands bound and transported by AGP, there exist two W catabolites—serotonin and melatonin—that are functionally destined to the central nervous system and the pineal gland, respectively. The other major APR is CRP, an unglycosylated pentameric peptide that is poorly detectable in plasma of healthy people. Its values are usually stagnant below 5 mg/L but may rise within 2–3 days by about 1000-fold or more in cases of acute inflammation in humans [69,73]. During its synthetic process, each CRP molecule must acquire five novel W residues to fully express its inflammatory potency. Most staple crops usually consumed in developing countries contain very low EAA levels, resulting in reduced (Trp) W bioavailability and stunting children’s development in African countries [74]. Because the dietary W shortage seems insufficient to meet CRP synthetic requirements, it is likely that the main source to fulfill these needs is the reservoir offered by ALB storage sites. Such (Trp) W withdrawal from ALB reserves might well result in protracted disease and undermine the recovery capacity of the malnourished and infected body.

### 5.4. Fe-Depleted Patients Reveal Declining Plasma W Values Correlated to Iron Indices

Hemoglobin (HGB) and transferrin (TF) work as two emblematic biomarkers that deserve to be mentioned among many other ferric components in the disturbances affecting iron metabolism in stress disorders. HGB is a tetrameric molecule containing 4 subunits [70] each with 2α- and 2ß-chains totaling 574 AAs, including 6 W residues. Each α-subunit bears one W residue (W^14^), and each ß-chain conveys two W residues (W^15,37^). HGB has a low W/AA ratio of 0.53, suggesting a relatively attenuated aptitude to face cytokine-induced stimuli. Conversely, as the normal human blood volume contains about 130 to 150 g/L of HGB, this constitutes a large disposal of W molecules. Under conditions leading to severe cases of iron deficiency, HGB levels may decline to 70–80 g/L, showing that about one half of its storing potential remains available for implementing further W needs. TF is a monopeptide possessing two glycan chains [71] and a sequence of 678 Aas, among which are 8 W residues yielding a slightly raised W/AA score (1.18). In field studies, a declining TF value is usually measured as an index of poor protein nutritional status [75]. A low TF receptor is currently used as a reliable marker of depressed hematopoiesis [76] that must therefore be adjusted to AGP and CRP values to take into consideration the infectious context. This corrective approach is partially invalidated in the case of coexisting iron deficiency that stimulates TF synthesis in opposite direction [75]. Recent clinical studies are striving to disentangle the pathophysiological mechanisms whereby reduced bioavailability of W may lead to alterations in iron and immune responses. An Austrian survey, undertaken on 115 iron-deficient patients revealed that declining W values were positively correlated with HGB and other markers of iron metabolism [77]. An Italian investigation, performed on 125 stroke subjects, revealed that reduced W levels were positively correlated with depressed ALB and HGB concentrations, with a tendency showing the lowest W levels in stroke patients suffering from the worst nutritional states [78]. A third clinical inquiry was performed on 105 low-income inhabitants of Pretoria (South Africa) consuming maize meal as staple food and who were taken into care for HIV infection and sometimes for co-morbidities (tuberculosis). The lowered plasma W values were positively correlated with ALB and HGB, disclosing inverse relationships with inflammatory indices (interleukin-6, CRP, and neopterin) [79]. Taken together, the collected data show that differences in gender, age, ethnicity, dietary and cultural habits, nutritional states, morbidities and adjunct disorders must be regarded as contingent features that should not depart from the basic connection linking W deficiency to ALB and HGB parameters. The binding interactions between hemin and serum ALB and HBG have been clarified using fluorescence quenching analysis, showing that the participation of both W and tyrosine is mandatory to acquire non-covalent W binding on the protein surface [80]. The quenching methodology entails complex conformational changes in hemoglobin structure (loosening and unfolding skeleton) but with an unaltered and functionally preserved heme structure [81].

### 5.5. A Scoring Formula Comprising the Main APRs Allows US to Tackle Stress Responses

The prognostic inflammatory and nutritional index (PINI) [82] is a ratio between the two most reliable indices of any inflammatory state (AGP and CRP) that are recommended by the WHO [33,83]. These indices constitute the numerator, being confronted to the two most trustworthy biomarkers of global protein status (TTR and ALB). The latter are narrowly correlated with LBM and compose the denominator of the PINI formula, as initially proposed by Ref. [82]. In healthy subjects, the PINI is neutral and close to 0, but it constitutes a balanced assessment between both the inflammatory and nutritional states since each product of the ratio comprises two slowly moving proteins (AGP and ALB), which may buffer the sometimes rapid fluctuations generated by CRP and TTR. The formula is beneficially utilized on large scales in Western children [84,85], in adults with several morbidities [86,87,88,89,90,91] and neoplastic disorders [92,93,94], and in elderly hospitalized subjects [95]. Moreover, the PINI formula has been adopted in field studies by African researchers working in Ivory Coast [96,97] and in Congo-Brazzaville [98]. A recent detailed investigation undertaken with hemodialysis patients highlighted several aspects of its clinical usefulness [99]: Although RBP does not structurally pertain to the PINI tetramer, there exists a strong likelihood that this retinol carrier protein actively participates in the W outburst during inflammatory processes due to its narrow proximity with TTR. We here recall that both TTR and RBP molecules are synthesized and coalesce into the liver parenchymal cells before undergoing extrahepatic secretion and plasma transport within a triplet stoichiometry 1:1:1 [23]. During the acute phase of inflammation, both biomarkers undergo simultaneous decay, releasing their hormonal ligands in free form [55], hence providing substantial W fluxes into the core of the hot spots.

## 6. W Catabolism: Disparity of Immune Responses and Specific Therapeutic Approaches

This section highlights the fact that dietary W deficiency should be recognized as a nutritional hot spot of major public health problems harming mankind. Our review collects the main nutritional and endocrine effects of W deficiency found in vulnerable inhabitants living in low-income areas vs. the alterations generated by W catabolites afflicting adult and elderly subjects from affluent societies.

### 6.1. Specificities of W Metabolism in Developing Countries

Most authors agree with data indicating that in developing countries, mainly in Africa, most edible cereals and roots are lacking in EAAs [74], principally W and lysine [100]. In the southern part of Congo where staple food consists of maize and cassava, a field study identified “extremely low W values” in plasma and urine of KW children [101]. The problems created by imbalanced protein and energy intake, together with W deficiency, are largely underestimated. Most restoration attempts performed in many countries are characterized by mitigated results showing the persistence of BW retardation, organ dysfunctions, and failure to improve protein, retinoid, and iron deficiencies with the risk of complications or relapses [102,103,104]. Ready-to-use therapeutic food (RUFT), ensuring the provision of rehabilitation formulas based on varying proportions of cereals, cannot be recommended due to their inability to sustain appropriate recovery of malabsorption capacities of the intestinal mucosa together with impairment of most immune properties. During the post-weaning period, maternal milk should be replaced with bovine milk, constituting a valuable, although not optimal, surrogate. It is here necessary to recall that the first epidemiological report written seventy years ago by WHO experts [12] correlated the best health states with local children growing in livestock farming areas. Severely protein-depleted infants may reveal at hospital entry TTR and RBP plasma values stagnant at 30% of normalcy [105]. After the instauration of inclusive bovine skimmed milk, these children displayed a curvilinear increase in both biomarkers, requiring only 3 weeks of refeeding before resuming normal levels [105], while their main functional capacities were substantially restored. In the case of intervening superinfection, both W-rich TTR and RBP undergo cytokine-induced decay, whereas increasing the synthesis of CRP and AGP monopolizes W stores, which are henceforth derived and no longer engaged in repair processes. The addition of dietary W supplementation should constitute a novel health measure of meaningful importance during the duration of infant recovery, which should stretch over (extend from) the weaning period until school age. The earliest campaign initiated by Tessema et al. in Ethiopia should serve as a model for all field workers facing comparable W deficits comparable to those of concern observed around the world [100]. The future administration of nutritional W shares similarities with intervention strategies ensuring the provision of iodine to goitrous patients [106]. Before administering W medicines to vulnerable population groups, it will be necessary to identify the most severely W-depleted areas. This is important because W may already confer preventive immune protection to clinically unrecognized malnourished patients [53]. The PINI score has a major impact on the follow-up of convalescent children, allowing us to determine the evolution of protein depletion and/or inflammatory burden.

### 6.2. Specificities of W Metabolism in Brain Disorders

Arising complications are different at the other side of the human lifespan, with most of them generated along the Kyn pathways [1,2], causing a number of health problems agglomerated around dual major domains, i.e., neurodegenerative disorders [9], with Alzheimer’s disease (AD) as the emblematic representative, and/or towards several types of neoplasia affecting most functional organs of the human body, such as acute myeloid leukemia [107], Hodgkin lymphoma [108], liver carcinoma [109], breast [110], lung [111], colorectal [112], and pancreatic tumors [113]. Other pathophysiological mechanisms have been deciphered in further detail in recent reviews [1,2] and intense efforts are currently being made by pharmacologists to inhibit the enzymatic defects implicated in these acquired metabolic defects. TTR is recognized as playing key roles in these neurodegenerative disorders, starting from two distinct production sites, namely the liver and choroid plexus (CP), whose functional roles are regulated independently [114]. The discovery that the TTR molecule may be converted into intracellular amyloid-ß oligomers has launched in-depth AD studies [115]. Further, the discovery that Aß toxic amyloid plaques may be neutralized by TTR [116] has given impetus to a large number of research advances marked by bumpy trajectories that may be, two decades later, briefly summarized as follows: TTR interacts with Aß-_40_ and Aß-_42_ oligomers, leading to protective effects through the inhibition of the primary and secondary nucleation processes, hence limiting the toxicity of both Aß oligomers [117] and sustaining fibril disruption [118]. Numerous plasma and CSF biomarkers are currently being scrutinized (examined) by neuropathologists to ensure the best follow-up of these neural disorders. Some research teams are defending the clinical usefulness of the neurofilament light chain (NfL) value correlated with Kyn metabolic derivatives [119]. Others are proposing the measurement of the Aß_42_/Aß_40_ ratio together with the NfL value and their association with total tau (T-tau) and phosphorylated tau (P-tau) in CSF and plasma [120]. Some researchers have reviewed the literature in terms of W plasma and CSF values with regard to Kyn metabolites [121]. No clear evolutionary patterns have emerged up to now from these multiple attempts to reach a unified spectrum of neural diseases, owing to the multiplicity and complexity of the pathophysiological factors participating in neurodegenerative disorders, among which prevail the stage of disease, as well as gender, age, genomics, nutritional /inflammatory states, and co-morbidities.

With the notable exception of TTR, most other proteins harbored in the CSF originate enter via BBB transfer across the choroid plexus epithelium. The transit mechanism is enabled by the cellular BBB specificity of the blood–CSF barrier, which responds to normal ALB signaling [122]. Trace amounts of other plasma proteins such as RBP [123], AGP [124], and CRP [125] may be identified in the intrathecal fluid after having crossed the blood–CSF barrier. In mammal species, the CSF/plasma ratio is very low, ranging usually between 1% and 3% for human ALB [122]. It is also noteworthy to underline that the transfer of plasma proteins through the BBB reaches its highest intrathecal concentration in the postnatal period to undergo progressive downregulation with increasing age [126]. The sluggish plasma and intrathecal responses recorded for nutritional biomarkers and inflammatory APRs likely result from the exhaustion of body protein and energy resources in protracted sickness. As an effect, the reduced resilience capacity explains why the PINI scoring formula is poorly informative in long-lasting neurodegenerative illnesses. The combination of protein malnutrition and neurodegenerative disorders results in a worse and likelier lethal outcome [127]. In this context, the measurement of plasma TTR should not be ignored as it remains the sole persisting biomarker allowing the faithful identification of LBM declining values [29,128].

## 7. Conclusions

Both clinical and biomedical data jointly provide a global overview of our current knowledge on the behavior of (Trp) W metabolism in health and disease. These two facets of disease appear to be complementary although marked by distinct responses taking into account geographical specificities, anthropocentric characteristics, and socio-cultural factors. Whereas daily provision of (Trp) W-enriched meals should be promoted in people with such depletions as a basic preventive nutritional measure, the therapeutic administration of inhibitory drugs reducing the harmful effects exerted by dysregulated key enzymes all along the (Trp) metabolic pathways has already displayed promising results in adults [1,2].

## Figures and Tables

**Table 1:** Characteristics of some mediators of energy and growth processes.

Denomination	AA mol.	AA	Trp	Ratio	Ref.
	Mass (kDa)	Residues	Residues	W/AA	
HGH	21	191	1 (W^25^)	0.52	[48]
IGF-1	7.6	70	0	0	[50]
Insulin	5.8	51	0	0	[49]
Glucagon	3.4	29	1 (W^25^)	3.45	[51]

**Table 2:** Molecular aspects of components involved in immune processes.

	Biomarker	Conformation	Molecular	AA Sequence	Trp Residues	Ratio W/AA	Biological (T_1/2_)	Ref.
			Mass (kDa)					
NUTRITION BIOMARKERS	TTR	Tetrameric (4 subunits)	55	508 (4 × 127)	⑧ 4 × W^41,79^	1.57	2 days	[20]
	RBP	Monopeptide	21	182	④ W^24,67,91,105^	2.19	14 h	[21]
	ALB	Polypeptide	66.5	585	① W^214^	0.17	19 days	[61]
ACUTE- PHASE REACTANTS	AGP	Peptide~58% & glycosides~42%	41–43	183	③ W^25,122,160^	1.64	3–5 days	[68]
	CRP	Pentameric monopeptide	21.5	187	⑤ W^81,91,142,168,186^	2.67	variable	[69]
IRON STATUS ANALYTES	HGB	Tetrameric (2 α chains + 2 ß chains)	64	574 (α: 282, ß: 292)	⑥ α: W^14^, ß: W^15,37^	1.04	20 days	[70]
	TF	Monopeptide (2 glycan chains)	79.5	678	⑧ W^8,128,264,344^ ^358,441,460,549^	1.18	8–10 days	[71]

## Data Availability

The review was generated using a large yearly compilation of WHO bibliography, PubMed resources, and *Nutrients* research data policies.
